# RETRACTION: MiR‐223 Promotes Tumor Progression via Targeting RhoB in Gastric Cancer

**DOI:** 10.1155/jo/9798674

**Published:** 2025-11-06

**Authors:** Journal of Oncology

RETRACTION: Y. Hu, B. Yi, X. Chen, L. Xu, X. Zhou, and X. Zhu, “MiR‐223 Promotes Tumor Progression via Targeting RhoB in Gastric Cancer,” *Journal of Oncology*, no. 2022 (2022), https://doi.org/10.1155/2022/6708871.

The above article, published online on 6 January 2022 in Wiley Online Library (https://wileyonlinelibrary.com), has been retracted by John Wiley & Sons Ltd.

The retraction has been agreed following an investigation of the concerns raised by *Actinopolyspora Biskrensis* on PubPeer [1], which identified multiple instances of figure duplication.

Specifically:

Figure 2a: The image of MGC803 cells treated with an NC-inhibitor shares overlapping features with the Blank Control image.

Figure 2b: Three panels of the wound healing assay are identical to the panels in Figure 2b of [2], where they depict different cells and experimental conditions.

Figure 2d: The apoptosis rate of MGC803 cells treated with an miR-223 inhibitor has unexpected similarities with Figure 2g (left) of [3], which shows the apoptosis rate of NSCLC cells. Additional concerns have also been identified regarding the similarity of the NC-inhibitor blot with the flow cytometry analysis of MCF-7 cells with 40 μM of nanoparticles in Figure 2b of [4].

As a result of the investigation, the data and conclusions of this article are considered unreliable.

The authors have been informed of this retraction but did not provide a response.
